# Supplementary material for: Resolving the full spectrum of human genome variation using Linked-Reads
Source: Genome Res. 2019 Apr;29(4):635–45. doi: 10.1101/gr.234443.118 (PMC6442396; doi:10.1101/gr.234443.118)
Supplement: Supplemental Material [file supp_gr.234443.118_Supplemental_longranger-2.2.zip › longranger-2.2.tar.bz2!/lr2.2-public/lib/go/src/code.google.com/vitess/docs/user-guide/vitess-replication.html]

Vitess / Vitess and Replication


Toggle navigation

- Overview
- Guides
- Reference
- Blog
- GitHub

- #### Overview

  - What is Vitess
  - Scaling MySQL with Vitess
  - Key Concepts
- #### Getting Started

  - Run Vitess on Kubernetes
    - Custom Docker Build
  - Run Vitess Locally
- #### User Guide

  - Introduction- Client Libraries- Backing Up Data- Reparenting
        - Schema Management
        - VSchema Guide
        - Schema Swap (Tutorial)
        - Sharding
          - Horizontal Sharding (Tutorial, automated)
          - Horizontal Sharding (Tutorial, manual)
          - Sharding in Kubernetes (Tutorial, automated)
          - Sharding in Kubernetes (Tutorial, manual)
        - Vitess Sequences
        - MySQL Server Protocol
        - Vitess and Replication
        - Update Stream
        - Row Based Replication
        - Topology Service
        - Transport Security Model
        - Launching
          - Scalability Philosophy
          - Production Planning
          - Server Configuration
          - 2PC Guide
          - Troubleshooting
        - Upgrading
- #### Reference Guides

  - Vitess API- vtctl Commands
- #### Other Resources

  - Presentations- Blog- Roadmap
- #### Contributing

  - Contributing to Vitess- GitHub Workflow- Code Reviews
- GitHub

# Vitess and Replication

- #### Overview

  - What is Vitess
  - Scaling MySQL with Vitess
  - Key Concepts
- #### Getting Started

  - Run Vitess on Kubernetes
    - Custom Docker Build
  - Run Vitess Locally
- #### User Guide

  - Introduction- Client Libraries- Backing Up Data- Reparenting
        - Schema Management
        - VSchema Guide
        - Schema Swap (Tutorial)
        - Sharding
          - Horizontal Sharding (Tutorial, automated)
          - Horizontal Sharding (Tutorial, manual)
          - Sharding in Kubernetes (Tutorial, automated)
          - Sharding in Kubernetes (Tutorial, manual)
        - Vitess Sequences
        - MySQL Server Protocol
        - Vitess and Replication
        - Update Stream
        - Row Based Replication
        - Topology Service
        - Transport Security Model
        - Launching
          - Scalability Philosophy
          - Production Planning
          - Server Configuration
          - 2PC Guide
          - Troubleshooting
        - Upgrading
- #### Reference Guides

  - Vitess API- vtctl Commands
- #### Other Resources

  - Presentations- Blog- Roadmap
- #### Contributing

  - Contributing to Vitess- GitHub Workflow- Code Reviews

# Vitess, MySQL Replication, and Schema Changes

## Statement vs Row Based Replication

MySQL supports two primary modes of replication in its binary logs: statement or
row based.

**Statement Based Replication**:

- The statements executed on the master are copied almost as-is in the master
  logs.
- The slaves replay these statements as is.
- If the statements are expensive (especially an update with a complicated WHERE
  clause), they will be expensive on the slaves too.
- For current timestamp and auto-increment values, the master also puts
  additional SET statements in the logs to make the statement have the same
  effect, so the slaves end up with the same values.

**Row Based Replication**:

- The statements executed on the master result in updated rows. The new full
  values for these rows are copied to the master logs.
- The slaves change their records for the rows they receive. The update is by
  primary key, and contains the new values for each column, so usually it’s very
  fast.
- Each updated row contains the entire row, not just the columns that were
  updated (unless the flag --binlog\_row\_image=minimal is used).
- The replication stream is harder to read, as it contains almost binary data,
  that don’t easily map to the original statements.
- There is a configurable limit on how many rows can be affected by one
  binlog event, so the master logs are not flooded.
- The format of the logs depends on the master schema: each row has a list of
  values, one value for each column. So if the master schema is different from
  the slave schema, updates will misbehave (exception being if slave has extra
  columns at the end).
- It is possible to revert to statement based replication for some commands to
  avoid these drawbacks (for instance for DELETE statements that affect a large
  number of rows).
- Schema changes always use statement based replication.
- If comments are added to a statement, they are stripped from the
  replication stream (as only rows are transmitted). There is a flag
  --binlog\_rows\_query\_log\_events to add the original statement to each row
  update, but it is costly in terms of binlog size.

For the longest time, MySQL replication has been single-threaded: only one
statement is applied by the slaves at a time. Since the master applies more
statements in parallel, replication can fall behind on the slaves fairly easily,
under higher load. Even though the situation has improved (parallel slave
apply), the slave replication speed is still a limiting factor for a lot of
applications. Since row based replication achieves higher update rates on the
slaves in most cases, it has been the only viable option for most performance
sensitive applications.

Schema changes however are not easy to achieve with row based
replication. Adding columns can be done offline, but removing or changing
columns cannot easily be done (there are multiple ways to achieve this, but they
all have limitations or performance implications, and are not that easy to
setup).

Vitess helps by using statement based replication (therefore allowing complex
schema changes), while at the same time simplifying the replication stream (so
slaves can be fast), by rewriting Update statements.

Then, with statement based replication, it becomes easier to perform offline
advanced schema changes, or large data updates. Vitess’s solution is called
schema swap.

We plan to also support row based replication in the future, and adapt our tools
to provide the same features when possible. See Appendix for our plan.

## Rewriting Update Statements

Vitess rewrites ‘UPDATE’ SQL statements to always know what rows will be
affected. For instance, this statement:

```
UPDATE <table> SET <set values> WHERE <clause>
```

Will be rewritten into:

```
SELECT <primary key columns> FROM <table> WHERE <clause> FOR UPDATE
UPDATE <table> SET <set values> WHERE <primary key columns> IN <result from previous SELECT> /* primary key values: … */
```

With this rewrite in effect, we know exactly which rows are affected, by primary
key, and we also document them as a SQL comment.

The replication stream then doesn’t contain the expensive WHERE clauses, but
only the UPDATE statements by primary key. In a sense, it is combining the best
of row based and statement based replication: the slaves only do primary key
based updates, but the replication stream is very friendly for schema changes.

Also, Vitess adds comments to the rewritten statements that identify the primary
key affected by that statement. This allows us to produce an Update Stream (see
section below).

## Vitess Schema Swap

Within YouTube, we also use a combination of statement based replication and
backups to apply long-running schema changes without disrupting ongoing
operations. See the schema swap tutorial
for a detailed example.

This operation, which is called **schema swap**, works as follows:

- Pick a slave, take it out of service. It is not used by clients any more.
- Apply whatever schema or large data change is needed, on the slave.
- Take a backup of that slave.
- On all the other slaves, one at a time, take them out of service, restore the
  backup, catch up on replication, put them back into service.
- When all slaves are done, reparent to a slave that has applied the change.
- The old master can then be restored from a backup too, and put back into
  service.

With this process, the only guarantee we need is for the change (schema or data)
to be backward compatible: the clients won’t know if they talk to a server
that has applied the change yet or not. This is usually fairly easy to deal
with:

- When adding a column, clients cannot use it until the schema swap is done.
- When removing a column, all clients must stop referring to it before the
  schema swap begins.
- A column rename is still tricky: the best way to do it is to add a new column
  with the new name in one schema swap, then change the client to populate both
  (and backfill the values), then change the client again to use the new
  column only, then use another schema swap to remove the original column.
- A whole bunch of operations are really easy to perform though: index changes,
  optimize table, …

Note the real change is only applied to one instance. We then rely on the backup
/ restore process to propagate the change. This is a very good improvement from
letting the changes through the replication stream, where they are applied to
all hosts, not just one. This is also a very good improvement over the industry
practice of online schema change, which also must run on all hosts.
Since Vitess’s backup / restore and reparent processes
are very reliable (they need to be reliable on their own, independently of this
process!), this does not add much more complexity to a running system.

## Update Stream

Since the SBR replication stream also contains comments of which primary key is
affected by a change, it is possible to look at the replication stream and know
exactly what objects have changed. This Vitess feature is
called Update Stream.

By subscribing to the Update Stream for a given shard, one can know what values
change. This stream can be used to create a stream of data changes (export to an
Apache Kafka for instance), or even invalidate an application layer cache.

Note: the Update Stream only reliably contains the primary key values of the
rows that have changed, not the actual values for all columns. To get these
values, it is necessary to re-query the database.

We have plans to make this Update Stream
feature more consistent, very resilient, fast, and transparent to sharding.

## Semi-Sync

If you tell Vitess to enforce semi-sync
(semisynchronous replication)
by passing the `-enable_semi_sync` flag to vttablets,
then the following will happen:

- The master will only accept writes if it has at least one slave connected
  and sending semi-sync ACK. It will never fall back to asynchronous
  (not requiring ACKs) because of timeouts while waiting for ACK, nor because
  of having zero slaves connected (although it will fall back to asynchronous
  in case of shutdown, abrupt or graceful).

  This is important to prevent split brain (or alternate futures) in case of a
  network partition. If we can verify all slaves have stopped replicating,
  we know the old master is not accepting writes, even if we are unable to
  contact the old master itself.
- Slaves of *replica* type will send semi-sync ACK. Slaves of *rdonly* type will
  **not** send ACK. This is because rdonly slaves are not eligible to be
  promoted to master, so we want to avoid the case where a rdonly slave is the
  single best candidate for election at the time of master failure (though
  a split brain is possible when all rdonly slaves have transactions that
  none of replica slaves have).

These behaviors combine to give you the property that, in case of master
failure, there is at least one other *replica* type slave that has every
transaction that was ever reported to clients as having completed.
You can then (manually,
or with an automated tool like Orchestrator)
pick the replica that is farthest ahead in GTID position and promote that to be
the new master.

Thus, you can survive sudden master failure without losing any transactions that
were reported to clients as completed. In MySQL 5.7+, this guarantee is
strengthened slightly to preventing loss of any transactions that were ever
**committed** on the original master, eliminating so-called
phantom reads.

On the other hand these behaviors also give a requirement that each shard must
have at least 2 tablets with type *replica* (with addition of the master that
can be demoted to type *replica* this gives a minimum of 3 tablets with initial
type *replica*). This will allow for the master to have a semi-sync acker when
one of the *replica* tablets is down for any reason (for a version update,
machine reboot, schema swap or anything else).

With regard to replication lag, note that this does **not** guarantee there is
always at least one *replica* type slave from which queries will always return
up-to-date results. Semi-sync guarantees that at least one slave has the
transaction in its relay log, but it has not necessarily been applied yet.
The only way to guarantee a fully up-to-date read is to send the request to the
master.

## Appendix: Adding support for RBR in Vitess

We are in the process of adding support for RBR in Vitess.

See this document) for more information.

Contact: vitess@googlegroups.com  **·**  
Announcements  **·**  
© 2017 Vitess powered by Google Inc
